# Facile synthesis of platinated DNA nanoparticles with radiosensitizing potential

**DOI:** 10.3762/bjnano.17.76

**Published:** 2026-08-13

**Authors:** Leo Sala, Tomas Perecko, Antonín Kaňa, Jaroslav Kočišek

**Affiliations:** 1 Dynamics of Molecules and Clusters Department, J. Heyrovský Institute of Physical Chemistry of the CAS, Dolejškova 3, Prague 182 23, Czech Republic; 2 Department of Cell Biology and Radiobiology, Institute of Biophysics of the Czech Academy of Sciences, Kralovopolska 135, 612 65 Brno, Czech Republic; 3 Department of Analytical Chemistry, University of Chemistry and Technology, Technická 5, 166 28 Prague, Czech Republic

**Keywords:** AFM, cisplatin, DNA nanostructures, radiosensitization

## Abstract

DNA nanostructures are promising drug delivery platforms but often suffer from limited structural stability and high production complexity. Cisplatin can cross-link DNA and act as a therapeutic agent, offering a simplified approach to stabilize DNA nanostructures while incorporating therapeutic functionality. Here, we explore cisplatin-mediated compaction of a single-stranded DNA scaffold to form stable nanoparticles and evaluate their potential as radiosensitizers. Cisplatin-induced cross-linking produced compact DNA nanoparticles with size and morphology controlled by mixing ratio and incubation time, reaching maximum loading near a 1:1 cisplatin-to-nucleotide mixing ratio, resulting in 0.28 ± 0.06 bound Pt atoms per nucleotide. The nanoparticles showed high thermal stability with the onset of thermal degradation between 80 and 90 °C and remained structurally stable at 4 °C storage for months. Clonogenic assays in FaDu cells demonstrated radiosensitization, with a dose enhancement ratio of 1.17 ± 0.07 at 10% survival. Cisplatin-mediated cross-linking provides a simple and effective method for producing stable DNA nanoparticles without complex origami assembly. The resulting structures retain the radiosensitizing properties of platinum while offering improved stability and storage characteristics. These platinated DNA nanoparticles represent a promising platform for further development of scalable DNA-based radiosensitizers with high drug loading, monodispersity, stability, and versatility for further functionalization.

## Introduction

DNA-based nanomaterials are widely investigated as drug delivery platforms [1–4] because of their inherent biocompatibility [5], low immunogenicity [6], high spatial addressability [7–8], and environmental responsiveness [9]. While DNA self-assembly techniques such as DNA origami allow for the precise arrangement of ligands and functional groups [10–11], these complex structures often suffer from rapid enzymatic degradation and structural dissociation in physiological environments [12–13]. These stability issues, occurring both in vitro and in vivo, remain a bottleneck preventing the transition from laboratory prototypes to viable clinical applications. Another issue preventing the bench-to-bed translation of DNA-nanostructure-based drug delivery systems is commercial feasibility. While the economics of DNA-based drug delivery systems benefit from novel nanostructure mass-production methods [14] and high achievable loading yields [15], the production complexity of sequence-based DNA nanostructure assemblies as well as regulatory issues persist [16–17].

Cisplatin serves as a primary chemotherapeutic agent for various malignancies [18], yet its clinical utility is frequently limited by high systemic toxicity [19–20] and development of tumor resistance [21]. Because the drug lacks a specific targeting mechanism, it is distributed broadly throughout the body, leading to significant off-target effects in healthy tissues. Effective sequestration of the drug within a delivery vehicle is essential to ensure that the payload is only released upon reaching the lower-pH or enzyme-rich tumor microenvironment [22–23]. DNA nanostructures are highly suitable for this purpose. Our prior research demonstrated that cisplatin can perform a dual role by acting as both the therapeutic payload and a stabilizing cross-linker that reinforces the DNA matrix [24].

Beyond its conventional role in chemotherapy, cisplatin acts as a potent radiosensitizer [25–26] by reacting with low-energy secondary electrons generated during radiotherapy to amplify DNA damage within tumor cells [27–28]. While this dose-enhancement effect is well characterized for free cisplatin [29], the translation of this synergy into DNA-based delivery systems is not yet established. This study investigates whether cisplatin-loaded DNA nanoparticles retain their radiosensitizing efficacy when sequestered within a nanostructured vehicle. Furthermore, this research provides a foundation for exploring the radiotherapeutic potential of other DNA-templated metallized nanostructures [30–31].

Moreover, for situations that do not require intricate shapes (strict distance-dependence of nanostructure decoration), DNA origami may not be the most cost-effective system. Instead of assembling intricate folded structures, we thus utilize only the scaffold and induce compaction through cisplatin-mediated cross-linking. This method produces stable nanoparticles while significantly reducing the amount of synthetic “staple” strands required, thereby lowering production costs. We have optimized the synthesis by systematically varying cisplatin concentration and incubation periods to achieve maximum loading efficiency. Furthermore, we demonstrate the robustness of these nanostructures, confirming both their thermal stability and long-term shelf life in unbuffered environments.

## Experimental

### Nanoparticle synthesis

Solvent exchange to H_2_O was first performed on a 100 nM, 100 μL p7249 scaffold supplied in 10 mM Tris buffer with 1 mM EDTA (Tilibit nanosystems) by centrifugal filtration through 100 kDa molecular weight cut-off Amicon filters three times, each time adding 400 μL of Milli-Q water (Millipore, USA). This stock solution was then mixed with the appropriate concentration of cisplatin solution to achieve the desired mixing ratios. The resulting mixture was then allowed to incubate at the desired reaction times without agitation. A final centrifugal filtration was performed to remove excess cisplatin and purify the platinated DNA nanoparticles. The DNA concentration of the mixtures was estimated by measuring their UV absorbance at 260 nm using a Denovix DS-11 FX+ spectrophotometer. The Pt content measured by ICP-MS was calibrated to the ratio of the absorbances at 230 and 260 nm (*A*_230_/*A*_260_) showing strong linear dependence above *A*_230_/*A*_260_ = 0.5 (Supporting Information File 1, Figure S3). This allows for a faster estimation of Pt loading of platinated DNA nanoparticles.

### Agarose gel electrophoresis

Gels with 1% agarose were prepared by dissolving 350 mg agarose (Serva) in 35 mL 0.5× TAE (Sigma-Aldrich) buffer with heating. A 3.5 μL drop of 10,000× SYBR Green I nucleic acid gel stain was added to the warm mixture and mixed thoroughly before pouring the gel in the cast. The gel was then allowed to solidy. The platinated DNA nanoparticle samples were diluted to 6 nM in a 10 μL solution. Two microliters of 6× DNA gel loading dye (Thermo Scientific) was then added to the solution and mixed properly before loading into the gel wells. The gel was then run at 100 V for 35 min at room temperature under 0.5× TAE buffer.

### Stability experiments at elevated temperatures and in serum-supplemented buffer

For stability tests at different temperatures, platinated DNA nanoparticle and pure ssDNA scaffold solutions (12 nM) were heated up to the desired temperature (23, 50, 60, 70, 80, and 90 °C) in a heating block and allowed to stay at that temperature for 6 min. The samples were then quickly moved to and stored in an ice bath (4 °C) before analysis. A microliter of the samples was used for atomic force microscopy (AFM) analysis, and the rest was used for agarose gel electrophoresis (AGE) analysis.

For stability tests in a serum-supplemented buffer, platinated DNA nanoparticle and pure ssDNA scaffold solutions were first buffer-exchanged into 1× phosphate-buffered saline (PBS; Carl Roth, pH 7.4) by three rounds of spin filtration using 100 kDa molecular weight cut-off Amicon filters. The final solutions were then prepared by adding fetal bovine serum (FBS; Gibco) to 10% (v/v), along with the appropriate amounts of 10× PBS stock and Milli-Q water to achieve a final composition of 1× PBS and a DNA concentration of 12 nM. Incubations were carried out at different time points ranging from 0 to 24 h, with the longest incubation initiated first to allow for simultaneous analysis by AGE. FBS was added only at the start of each respective incubation time point. AGE was subsequently performed to assess potential degradation of the DNA scaffolds and nanoparticle structures.

### Atomic force microscopy

Si substrates (∼7 × 7 mm^2^) were first plasma-cleaned using a Roplass RPS40+ apparatus. One microliter of the platinated DNA nanoparticle solution (12 nM) was then dripped onto the surface of the plasma-cleaned Si substrate and subsequently added with 15 μL of 10× TAE with 125 mM MgCl_2_. The resulting droplet was allowed to rest for at least 30 min on the surface before washing it with 1 mL of 50% ethanol solution and finally dried using N_2_ gas. Imaging was then performed in air using the Dimension Icon AFM (Bruker) PeakForce Tapping Technology and ScanAsyst probes (40 kHz, 0.4 N·m^−1^).

### Particle size distribution

Atomic force microscopy (AFM) images of the different samples, acquired over a scan area of 3 × 3 μm^2^, were first flattened using the Gwyddion software by applying the following preprocessing steps: automatic data leveling through mean-plane subtraction, row alignment using the median method, and horizontal scar correction. The minimum height value was set to zero, and the color scale was stretched to a range of 0–6 nm to enhance the contrast between the particles and the background. The processed images were then saved and analyzed in ImageJ for particle detection and area measurement. Each image was first converted to an 8-bit grayscale image, after which image segmentation was performed by adjusting the threshold to 70–255. Particles were automatically identified using the “Analyze Particles” function with the circularity parameter set to 0–1, while excluding particles that intersected the image edges. A binary mask was also generated to verify that all particles had been correctly detected. This procedure produced a table containing the projected area of each particle, calibrated in square nanometers according to the image resolution. The equivalent particle diameters were then estimated from the measured projected areas by assuming a circular particle geometry. Finally, a histogram of the calculated particle diameters was generated to obtain the particle size distribution.

### Inductively coupled plasma mass spectrometry

The inductively coupled plasma mass spectrometry (ICP-MS) analysis of Pt content in the samples was performed using a NexION 5000 spectrometer (PerkinElmer, USA). Pt stock solution (Astasol^®^, Analytika Ltd., Czech Republic) with a concentration of 1000 ± 2 mg·L^−1^ was used to prepare calibration solutions. All solutions were acidified with nitric acid (Analpure^®^ grade, Analytika Ltd., Czech Republic) and diluted with Milli-Q water (Millipore, USA).

### Clonogenic assay

FaDu (#305033, squamous cell carcinoma of the hypopharynx) cell line was acquired from Cytion (Germany). Cells were cultured in RPMI-1640 medium (#21875‑091, Gibco, USA) supplemented with 10% FBS (#10270106, Gibco, USA) and 1% penicillin/streptomycin (#LM-A4118/100, Biosera, France). Cells were maintained at 37 °C in a humidified atmosphere with 5% CO_2_. Cells were authenticated by the vendor using STR profiling, and all mycoplasma tests were negative. The FaDu cells (700 cells per well in 1.5 mL RPMI media with 10% FBS) were then plated in 6-well plates and allowed to adhere overnight. The cells were treated with either the p7249 scaffold or platinated DNA nanoparticles by replacing the cell medium with that containing 5 nM p7249 scaffold or platinated DNA nanoparticles (equivalent cisplatin concentration of 10 μM). The treated cells were allowed to incubate for 24 h and subsequently irradiated with gamma rays at a dose rate of 1 Gy·min^−1^ using a ^60^Co source (Chisostat, Chirana, Czech Republic). After the irradiation, the medium was replaced by fresh medium to wash off extracellular nanoparticles or scaffold DNA. After two weeks, the colonies were fixed, stained with sulforhodamine B, and then counted by taking photos of the wells using a camera (BASLER, type acA2440-35uc) and counting the colonies by binary image segmentation and particle analysis using the ImageJ software. Image acquisition and particle detection parameters are specified in Figure S4 (Supporting Information File 1). The survival fraction represents the number of colonies grown after each irradiation dose with respect to the unirradiated control within each treatment group. Irradiation was done at doses of 2, 4, and 8 Gy for two independent experiments for the platinated DNA nanoparticles and the scaffold DNA and three independent experiments for the control (cells only) treatment.

The survival fractions (SF, *n* = 2–3) were plotted as a function of dose and fitted to the linear-quadratic (LQ) model in Python 3.14.2 using the “scipy” package and Equation 1. The fitted α and β parameters, along with their corresponding standard errors, were obtained from the regression analysis and used to calculate the dose required to achieve 10% survival (*D*_10_) according to Equation 2. The dose enhancement ratio (DER) was subsequently calculated as the ratio between the *D*_10_ value for the control condition (cells only) and that of the corresponding treatment system (platinated nanoparticle or P7249 scaffold), as described in Equation 3. The uncertainty in *D*_10_ was estimated by propagating the standard errors of the fitted α and β parameters using the first-order error propagation method (Equation 4). The uncertainty in DER was calculated by propagating the uncertainties associated with the *D*_10_ values of the control and corresponding treatment systems according to Equation 5. The reported uncertainties represent the propagated standard errors of the LQ model fit parameters and provide an estimate of the precision associated with the calculated *D*_10_ and DER values based on the regression analysis. These uncertainties reflect the confidence in the fitted model parameters and their derived quantities.


[1]

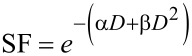




[2] $$D_{10}=\frac{-\alpha +\sqrt{\alpha^{2}-4\beta \ln\left(0.1\right)}}{2\beta }$$

[3] $$\text{DER}=\frac{D_{10,\text{control}}}{D_{10,\text{system}}}$$

[4] $$\sigma_{D_{10}}=\sqrt{{\left(\frac{\partial D_{10}}{\partial \alpha }\sigma_{\alpha }\right)}^{2}+{\left(\frac{\partial D_{10}}{\partial \beta }\sigma_{\beta }\right)}^{2}}$$

[5] $$\sigma_{\text{DER}}=\text{DER}\sqrt{{\left(\frac{\sigma_{D_{10,\text{control}}}}{D_{10,\text{control}}}\right)}^{2}+{\left(\frac{\sigma_{D_{10,\text{system}}}}{D_{10,\text{system}}}\right)}^{2}}$$

## Results and Discussion

**Figure 1:**
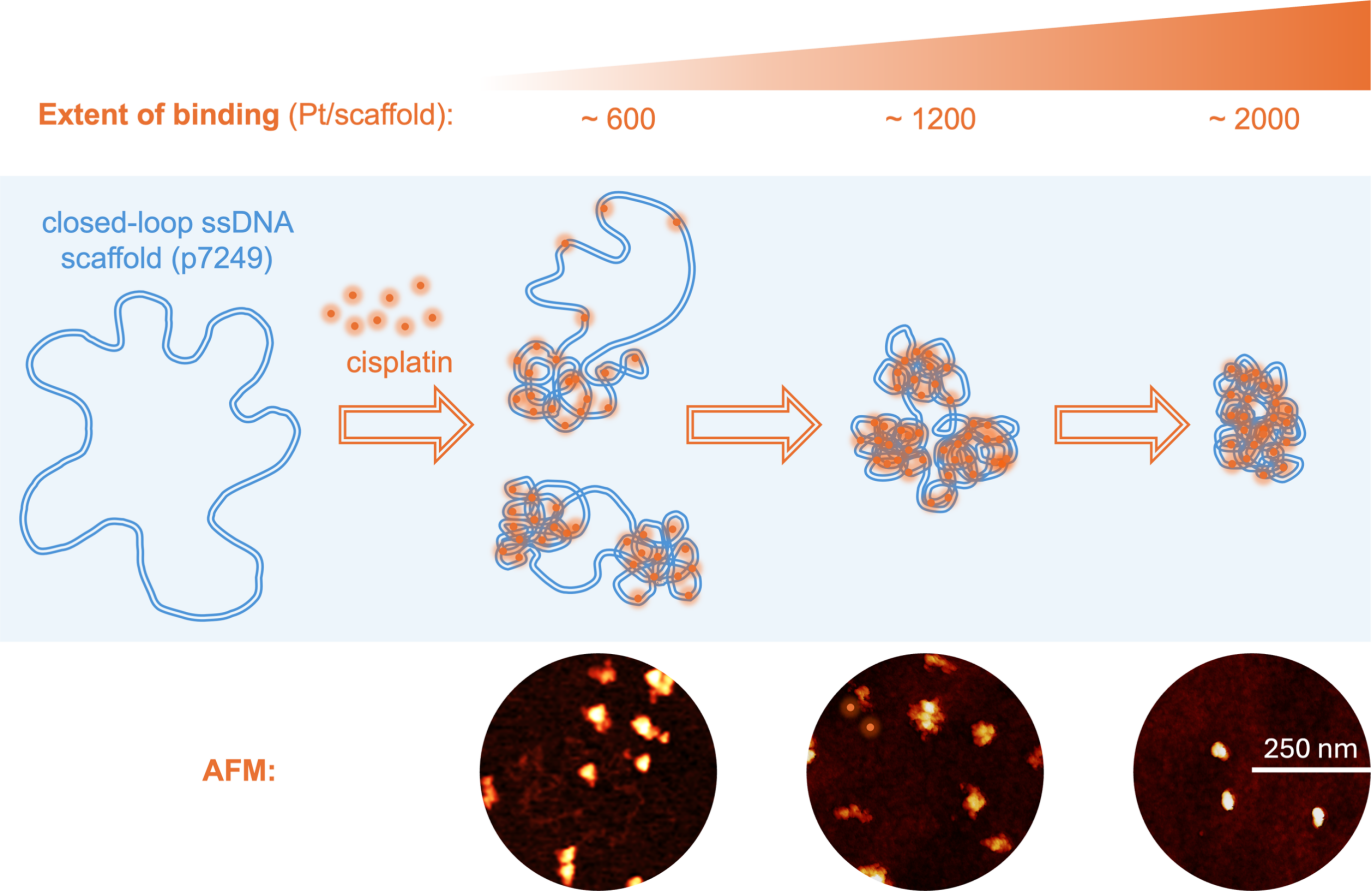
Schematic representation of the proposed platinated DNA nanoparticle assembly. A simplified model illustrates the compaction of the ssDNA scaffold driven by progressive cisplatin-mediated cross-linking, which could be initiated via localized loop formation. The upper panel displays the approximate number of cisplatin molecules bound per nanostructure. The lower panel shows representative AFM images; each circular crop has a radius of 250 nm for scale.

The nanoparticle design concept and the suggested assembly mechanism are illustrated in Figure 1. The approach is based on the controlled compaction of a circular single-stranded p7249 DNA (isolated from an M13mp18 lac phage vector) scaffold of defined length through cisplatin-mediated cross-linking. The circular ssDNA could be treated as a flexible polymer chain that can potentially undergo a progressive coil-to-globule transition as cross-links are introduced. Typically, the formation of intramolecular cross-links in polymers reduces chain entropy and promotes localized loop formation, which subsequently evolves into a compact, collapsed structure as the cross-link density increases. Utilizing a circular scaffold of fixed length constrains the conformational space of the polymer, which could result in narrower nanoparticle size distributions as opposed to approaches relying on short oligonucleotides or fragmented genomic DNA. This strategy could therefore enable controlled nanoparticle formation through a combination of polymer collapse and cross-linking-driven compaction. The next subsections describe the optimization of the parameters for the synthesis of the platinated DNA nanoparticles, driven by this design concept.

### Effect of mixing ratio

**Figure 2:**
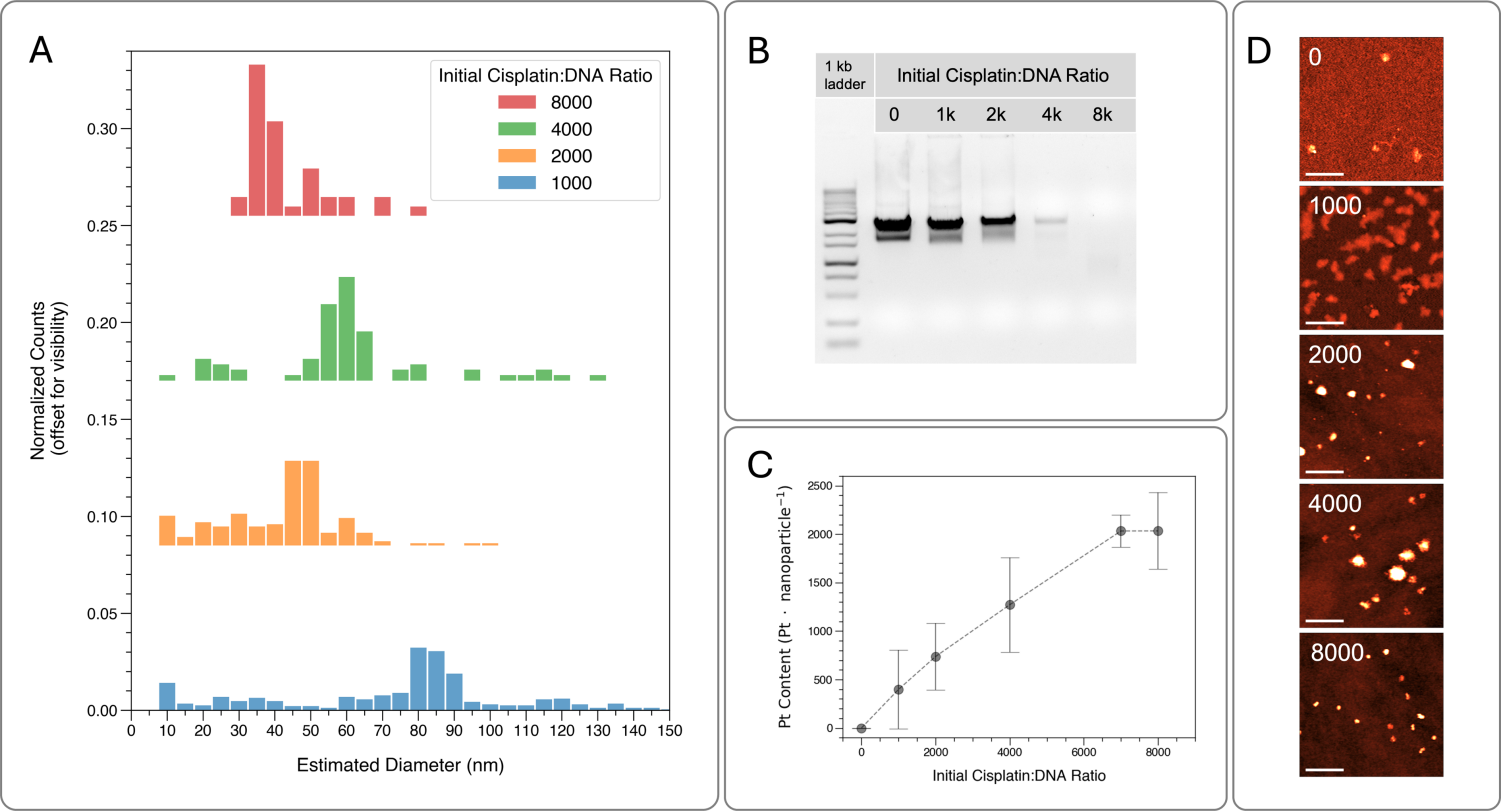
Size dependence on cisplatin concentration. Particle size distribution based on estimated diameters of particles detected in a 3 × 3 μm AFM image (A), AGE micrograph (B), Pt content (C), and representative 1 × 1 μm AFM images, scale bar = 250 nm (D) of platinated DNA nanoparticles synthesized with various cisplatin/DNA scaffold mixing ratios.

The initial cisplatin/DNA scaffold mixing ratios were explored by varying the cisplatin concentration while keeping the scaffold concentration (12 nM) and incubation time constant (16 h). The resulting samples were analyzed by AGE and AFM to determine particle size and distribution. The cisplatin content of purified samples was estimated using the ratio of UV–vis absorbances at 230 and 260 nm (*A*_230_/*A*_260_) calibrated against ICP-MS measurements of total Pt content of test solutions. Figure 2A shows the particle size distribution derived from 3 × 3 μm AFM images (representative cropped areas are shown in Figure 2D) of particles synthesized at different mixing ratios. A general reduction in particle size is observed as the cisplatin/DNA scaffold ratio increases. However, a transition occurs around a 4000 cisplatin/DNA scaffold ratio, where structures temporarily increase in size before becoming more compact again at 8000 cisplatin/DNA. Both AGE and AFM analyses reveal structural features that help explain this behavior.

In H_2_O, with only residual salts from DNA scaffold purification, the DNA scaffold exists primarily in a relaxed form, although collapsed or super-coiled structures arising from secondary interactions may also be present. In Figure 2B, these two forms are evident in the control lane (pure DNA scaffold): The relaxed structure appears as an intense broad band, while the super-coiled form appears as a fainter, higher-mobility band. Both structures are also visible in the AFM image of the control sample, although additional super-coiling may occur due to the presence of divalent salts required for deposition. At cisplatin/DNA scaffold ratios between 1000 and 4000, these bands remain visible. Since all samples were loaded at equal concentrations, the observed decrease in band intensity reflects cisplatin binding, which interferes with intercalation of the nucleic acid stain (SYBR Green I). At these ratios, no significant mobility shifts are observed. However, corresponding AFM images reveal taller and more compact structures, particularly at 2000 and 4000 cisplatin/DNA scaffold ratios.

Cisplatin cross-links seem to localize within specific regions of the scaffold, forming loop-like structures observed at the 2000 cisplatin/DNA scaffold ratio, while less extensively cross-linked segments remain relatively extended. AFM images of salt-free deposition on a mica substrate (Supporting Information File 1, Figure S6) better highlight these features. Similar formation of small loops has been reported in double-stranded DNA upon cisplatin loading [32]. The smaller apparent particle size distribution compared to the 4000 cisplatin/DNA scaffold ratio is also influenced by limitations in the particle detection method and image resolution, which cannot fully resolve the “free”, extended portions of the ssDNA scaffold. As a result, only the condensed looped regions are detected as discrete particles. At a 4000 cisplatin/DNA scaffold ratio, however, these localized loops already appear to occupy most of the scaffold, resulting in the formation of larger clumps. Some of these larger structures may also arise from intermolecular cross-linking between two or more scaffolds or from aggregation induced by substrate interaction or deposition conditions. Increasing the ratio further to 8000 leads to complete structural collapse into more cross-linked, compact nanoparticles. A sketch of the proposed mechanism of scaffold compaction by increasing cisplatin binding/cross-linking is shown in Figure 1.

The band corresponding to the 8000 cisplatin/DNA scaffold ratio in AGE (Figure 2B) is nearly invisible because of a variety of factors that can be associated with the reduced access of the nucleic acid stain through the compact nanostructure for intercalation and possible fluorescence quenching. This band is also observed at a higher mobility, consistent with a more compact structure compared to lower-loading variants. This trend is further supported by the particle size distribution shown in Figure 2A. Consequently, as seen in Figure 2C, maximum compaction and loading can be achieved at an cisplatin/DNA scaffold mixing ratio of approximately 8000, corresponding to 2000 ± 400 bound Pt atoms per nanostructure (0.28 ± 0.06 Pt atoms per nucleotide). The system where the mixing ratio was set to one cisplatin per nucleotide (7200 cisplatin/DNA scaffold mixing ratio) was evaluated; already under these conditions, the maximum loading is achieved after 16 h of incubation.

### Effect of incubation time

**Figure 3:**
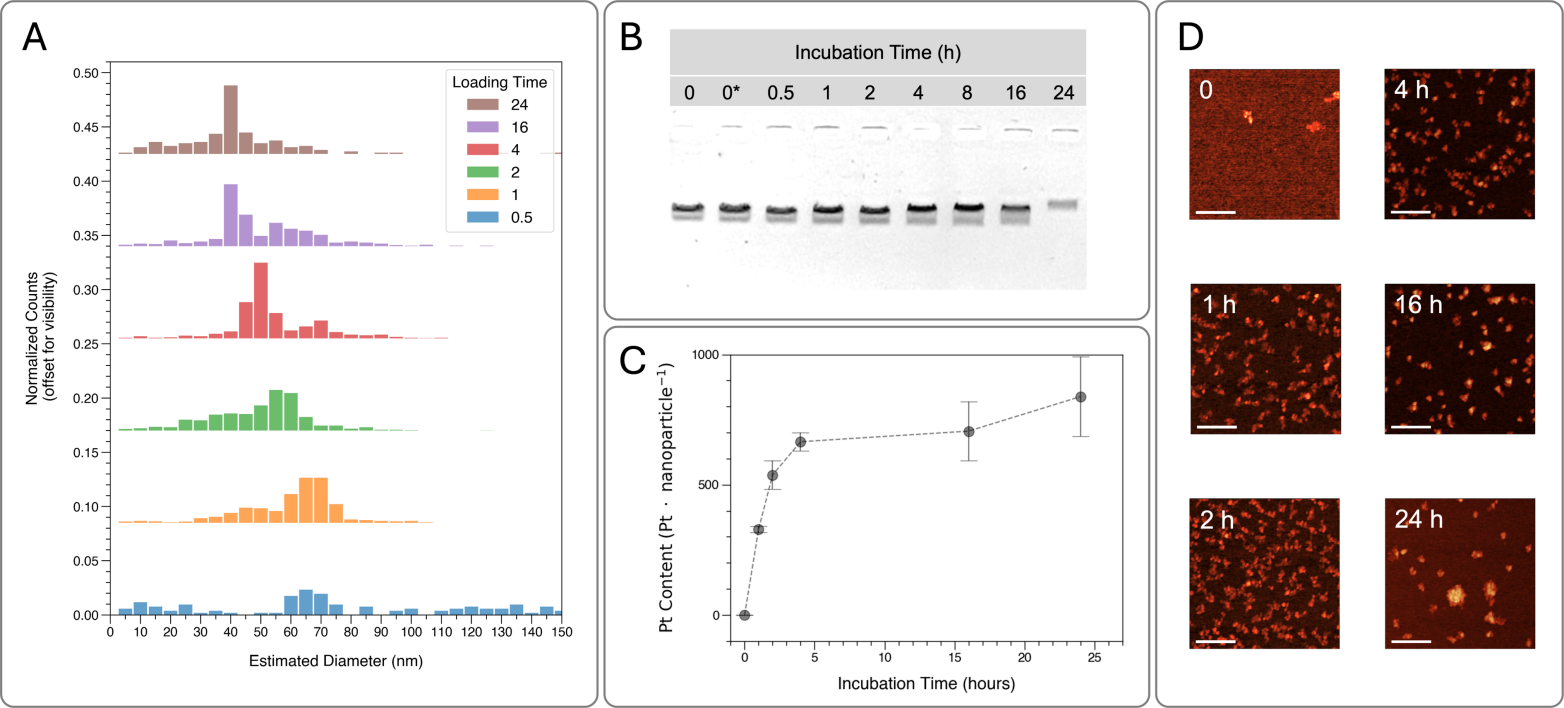
Size dependence on incubation time. Particle size distribution based on estimated diameters of particles detected in a 3 × 3 μm AFM image (A), AGE micrograph (B), and representative 1 × 1 μm AFM images, scale bar = 250 nm (C) of platinated DNA nanoparticles synthesized with different incubation times. In (B), the lane at incubation time 0 corresponds to the pure scaffold without the addition of cisplatin, while 0* represents the sample with cisplatin added immediately before loading into the gel well.

We used the 2000 cisplatin/DNA scaffold ratio to investigate the effect of incubation time on nanoparticle formation. This ratio was selected because the structures remain visible by gel electrophoresis, enabling easier monitoring, and because it lies below the saturation regime. As expected, compaction increased with incubation time, with particle size decreasing as more time was allowed for cisplatin binding (Figure 3A).

Formation of discrete nanoparticles began at approximately 4 h (Figure 3D), where the cisplatin uptake also begins to slow down (Figure 3C). At 16 h, the AGE results (Figure 3B) still show two bands corresponding to the supercoiled and relaxed states, indicating that the structures remain partially compacted, suggesting localized cross-linked regions and extended loops. After 24 h, further binding and compaction are observed, albeit some larger structures are present due to agglomeration either in solution or during deposition. It is also worth noting that they reproduce structures similar to those obtained at 4000 cisplatin/DNA scaffold ratio incubated for 16 h.

### Stability

**Figure 4:**
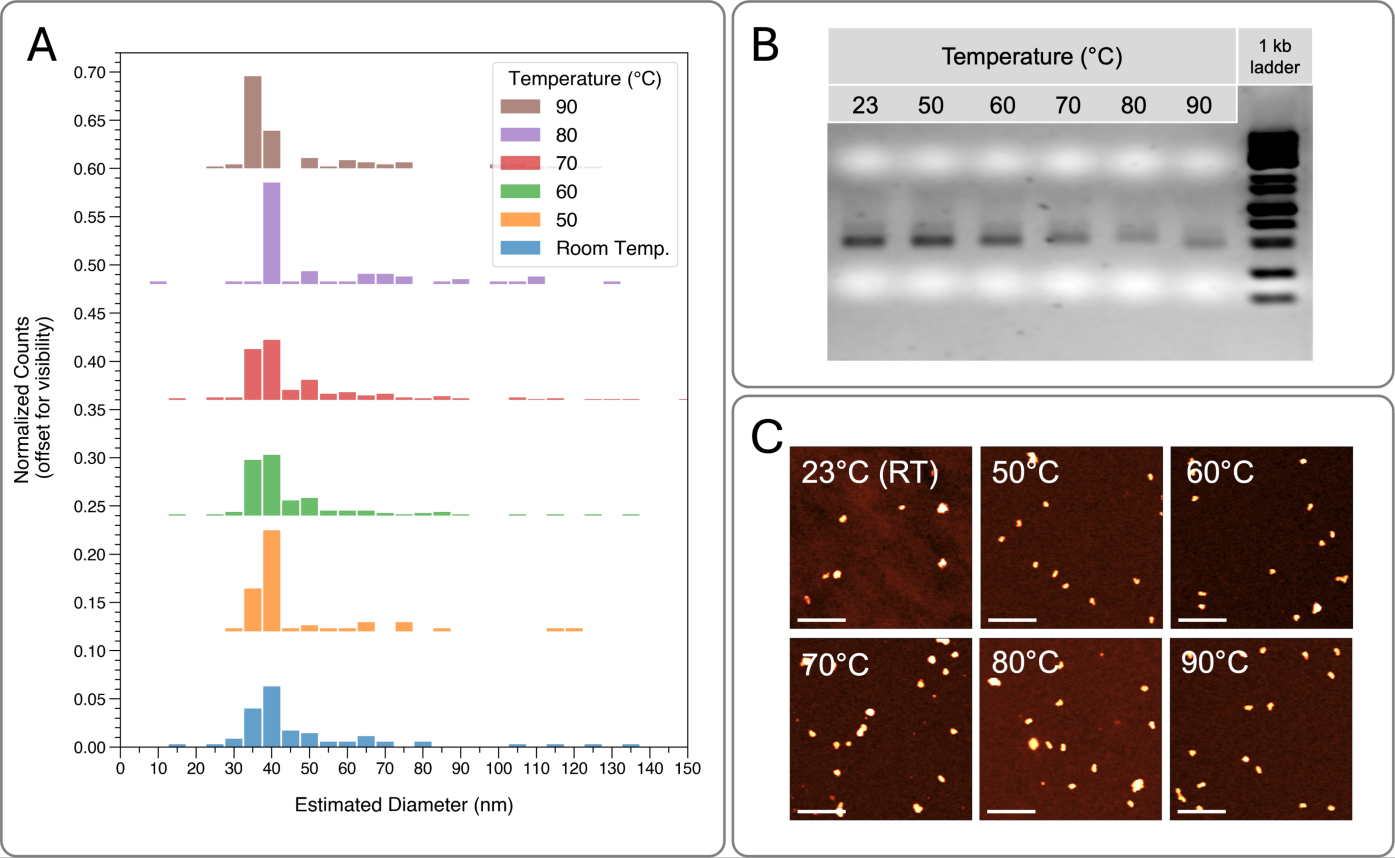
Thermal stability of platinated DNA nanoparticles. Particle size distribution based on estimated diameters of particles detected in a 3 × 3 μm AFM image (A), AGE micrograph (B), and representative 1 × 1 μm AFM images, scale bar = 250 nm (C) of platinated DNA nanoparticles heated to different temperatures from room temp to 90 °C.

To evaluate thermal stability, solutions of platinated DNA nanoparticles (prepared using 8000 cisplatin/scaffold mixing ratio and 16 h incubation time) were heated to temperatures up to 90 °C for 6 min and then quickly quenched in ice to minimize reassembly and drastic conformational changes. The particle size distributions obtained after heating are shown in Figure 4A with corresponding AFM images in Figure 4C. No significant changes in particle size were observed across the temperature range, indicating that the nanoparticles remain structurally intact under elevated temperatures for a few minutes. At 90 °C, however, the size distribution maximum appears slightly skewed toward smaller diameters. This shift may reflect either limitations in AFM resolution at smaller particle sizes or the onset of partial structural degradation. Consistent with this observation, AGE analysis shows a slight mobility shift for samples heated to 90 °C, suggesting minor structural changes at this temperature (Figure 4B). This shift may therefore represent the approximate threshold at which thermal degradation begins to occur.

For comparison, the unmodified DNA scaffold was exposed to the same heating conditions (Supporting Information File 1, Figure S1). In this case, AGE profiles reveal pronounced degradation already at 80 °C in unbuffered solution. The improved thermal stability of the platinated nanoparticles therefore indicate that cisplatin-induced cross-linking and compaction can stabilize the DNA structure up to a certain extent. This enhanced stability exceeds the one typically observed for conventional DNA nanostructures, which generally exhibit lower thermal tolerance under similar conditions [33].

Furthermore, we evaluated the stability of the platinated DNA nanoparticles under near-physiological conditions by incubating them in PBS (pH 7.4) supplemented with 10% FBS at 37 °C for 1–24 h, followed by analysis using gel electrophoresis (Supporting Information File 1, Figure S2). Compared with the free DNA scaffold, which shows evidence of degradation after 1 h of incubation, the gel band corresponding to the platinated DNA nanoparticles shows no detectable smearing or significant loss of intensity throughout the 24 h incubation period, indicating improved stability under these conditions. This could extend the application of the platinated DNA nanoparticles for in vitro and in vivo drug delivery explorations.

**Figure 5:**
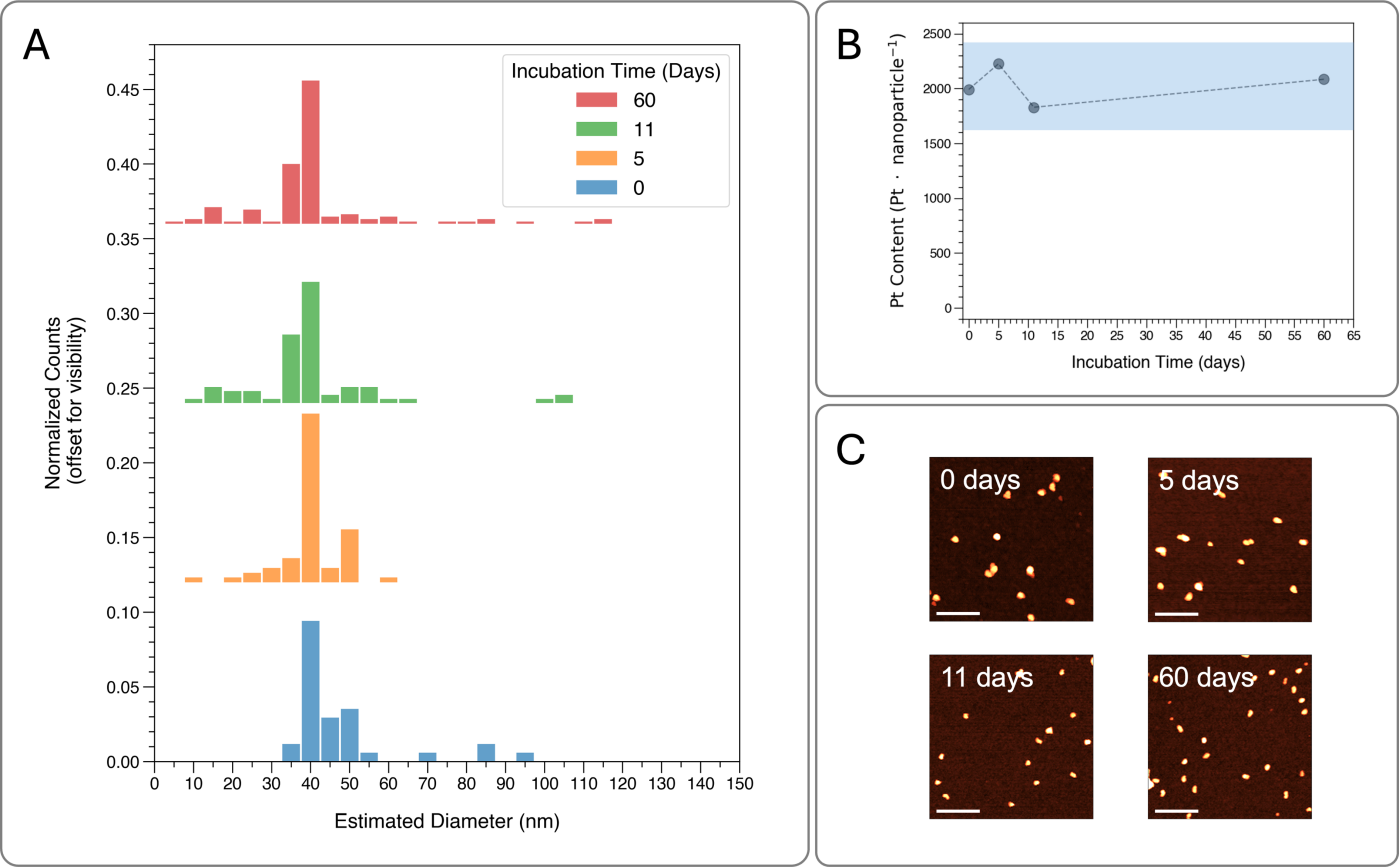
Long-term stability of platinated DNA nanoparticles in refrigerated storage. Particle size distribution based on estimated diameters of particles detected in a 3 × 3 μm AFM image (A), Pt content estimated UV–vis measurements (B), and representative 1 × 1 μm AFM images, scale bar = 250 nm (C) of platinated DNA nanoparticles stored at 4 °C for different durations. In (B), the uncertainty in Pt content measurements derived from UV–vis measurements as calibrated from ICP-MS measurements is shaded in blue.

We also evaluated the long-term stability of the nanoparticles by monitoring particle size and cisplatin content for samples stored in H_2_O at 4 °C over a period of two months. AFM analysis (Figure 5A and 5C) revealed minimal changes in particle size distribution throughout the storage period, indicating that the nanoparticles remain structurally stable under these conditions. In addition, the measured variation in cisplatin content remained within the uncertainty of the Pt quantification derived from UV–vis absorption measurements, suggesting negligible loss of bound cisplatin over time (Figure 5B). Collectively, these findings demonstrate good long-term stability of the platinated DNA nanoparticles during storage at 4 °C in aqueous solution, offering a practical storage advantage compared with conventional therapeutic agents.

### Radiotherapeutic potential

**Figure 6:**
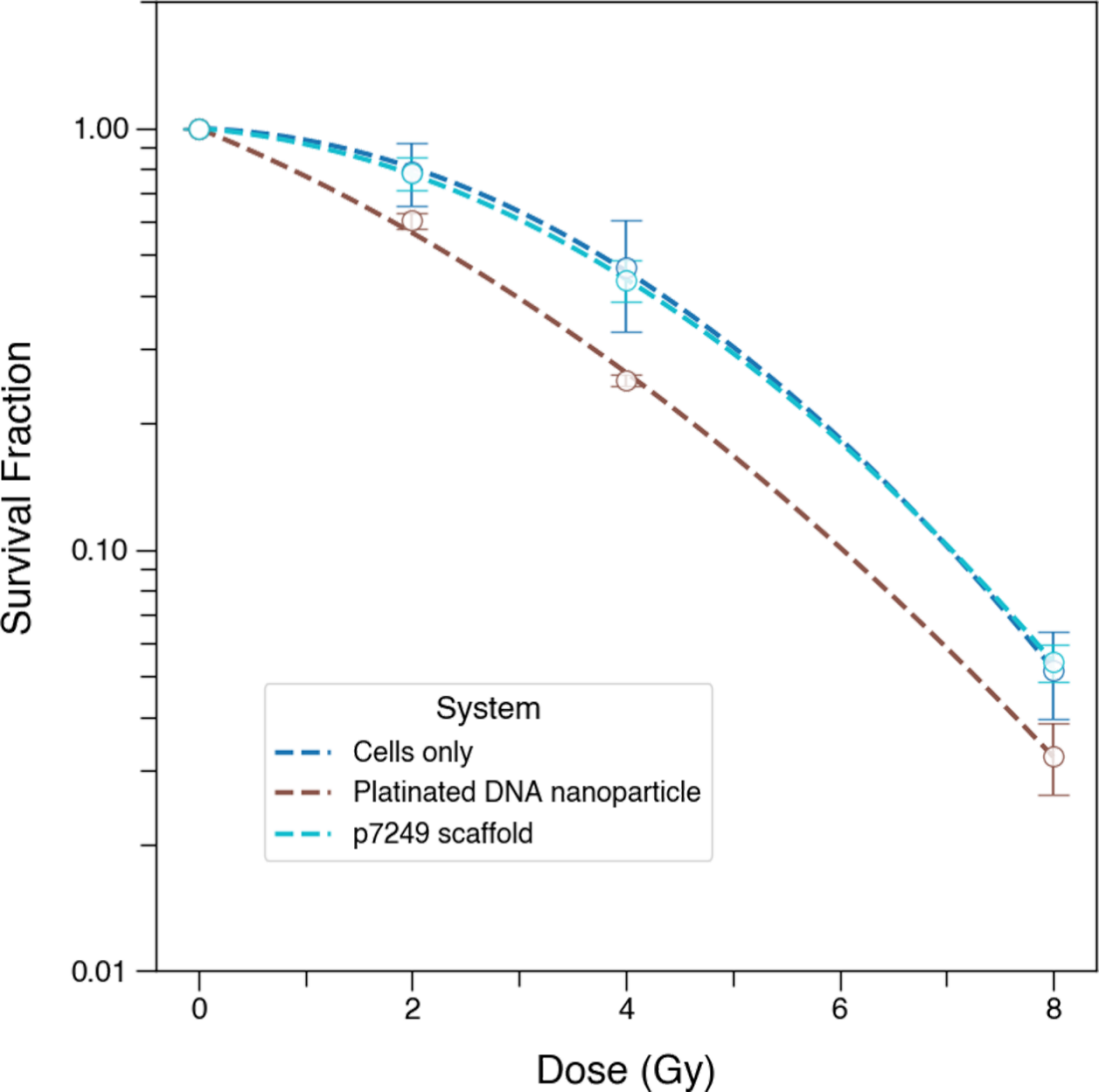
Clonogenic assay. Dose response of colony survival fraction (SF ± SEM) of FaDu cells incubated with platinated DNA nanoparticles (*n* = 2, brown), p7249 scaffold (*n* = 2, light blue), or medium only (*n* = 3, dark blue) for 24 h and then irradiated with gamma rays. Fits following the LQ model (Equation 1) are shown in dashed lines.

Platinum-based nanoparticles have been widely investigated as radiosensitizers in radiotherapy, motivating us to evaluate the potential of the platinated DNA nanoparticles in this context. A proof-of-concept radiosensitization assay was employed by using the FaDu cell line, a model for head and neck cancer. Survival fractions were plotted as a function of radiation dose (Figure 6). The resulting survival curves were fitted using the LQ model (Equation 1, Experimental section), where α and β represent the coefficients of the linear and quadratic components and are associated with direct (single-track) and indirect (cumulative) damages, respectively. From these fits, the dose required to achieve 10% survival *D*_10_ was determined, and the DER was calculated by comparing *D*_10_ values of treated samples to the cell-only control (Equation 2–Equation 3, Experimental section). The values are listed in Table 1.

**Table 1:** Parameters (α and β ± SEM) of the dose-response curve fits for the systems studied as well as the calculated effective dose (*D*_10_ ± SEM) and dose enhancement ratios at 10% survival (DER ± SEM).

Sample	α [Gy^−1^]	β [Gy^−2^]	*D*_10_ [Gy]	DER

control (cells only)	0.020 ± 0.009	0.044 ± 0.001	7.03 ± 0.13	1.00
p7249 scaffold	0.047 ± 0.003	0.040 ± 0.001	7.04 ± 0.05	1.00 ± 0.02
platinated DNA nanoparticles	0.238 ± 0.022	0.024 ± 0.003	6.03 ± 0.33	1.17 ± 0.07
cisplatin (0.2 μM)	0.0628 ± 0.003	0.045 ± 0.001	6.46 ± 0.04	1.09 ± 0.02

The *D*_10_ values obtained were 7.03 ± 0.13 Gy for the cell-only control, 7.04 ± 0.05 Gy for the free ssDNA scaffold, and 6.03 ± 0.33 Gy for the platinated DNA nanoparticles. The nearly identical *D*_10_ values for the control and free DNA scaffold indicate that the scaffold itself does not significantly influence radiosensitivity, confirming its suitability as an inert carrier. In contrast, the reduced *D*_10_ observed for the platinated DNA nanoparticles corresponds to a DER of 1.17 ± 0.07 compared to 1.00 ± 0.02 for the free ssDNA scaffold. This represents an enhancement in radiosensitivity of approximately 10–24%, considering the experimental uncertainty. The observed radiosensitization is consistent with the presence of platinum within the nanoparticles, which can increase local energy deposition and enhance radiation-induced DNA damage through secondary electron production and reactive species generation [34]. In FaDu cells irradiated with photon ionizing sources, DER values of approximately 1.20 have been reported for free cisplatin at micromolar concentrations [35]. We have also used the same assay on cisplatin concentrations with limited 10–20% reduction in the cell plating efficiency and observed about 9% DER at 0.2 μM (Supporting Information File 1, Figure S5). The comparable magnitude of radiosensitization observed here using 10 μM cisplatin incorporated within platinated DNA nanoparticles indicates that platinum retains its radiosensitizing functionality when embedded within the DNA nanostructure, while simultaneously benefiting from the structural stability and potential delivery advantages of the nanoparticle platform in this particular cell line and under these irradiation conditions. This modest enhancement may have been influenced by the free, uncross-linked domains acting as radical scavengers, though the nearly identical responses observed in the pure scaffold DNA and control groups suggest this effect was minimal. Additionally, because the cell media were replaced only after irradiation, the observed outcomes likely reflect a combination of both extracellular and intracellular release. Future studies would benefit from exploring cellular uptake, intracellular localization, and the impact of the limited release of coordinately bound cisplatin, all of which may constrain the full radiosensitizing potential of these nanoparticles. Further optimizations could be achieved by tuning nanoparticle concentrations, extending pre-irradiation incubation times to enhance uptake, or employing more active release mechanisms. Nonetheless, these findings indicate that platinated DNA nanoparticles show potential as radiosensitizers and warrant further optimization and systematic investigation.

## Conclusion

We demonstrated a simple approach for the synthesis of platinated DNA nanoparticles based on cisplatin-mediated cross-linking of a single-stranded DNA scaffold, which could be potentially scalable due to the use of only a few starting materials. By systematically varying cisplatin-to-DNA scaffold ratio and incubation time, we identified the conditions required for efficient nanoparticle formation and cisplatin loading. Increasing the cisplatin concentration promoted progressive scaffold compaction, with fully collapsed nanoparticles forming near a 1:1 cisplatin-to-nucleotide ratio, representing a remarkable drug load content for the carrier nanoparticle. Time-dependent experiments appeared to be consistent with a nanoparticle formation process involving initial localized cross-linking and eventual scaffold compaction, providing insight into the mechanism of cisplatin-induced DNA scaffold collapse.

The resulting platinated DNA nanoparticles exhibited high thermal stability for a DNA-based material, maintaining structural integrity up to 80 °C, with only minor indications of degradation at the highest temperature tested (90 °C). In contrast to the free DNA scaffold, the platinated DNA nanoparticles showed no degradation after 24 h of incubation in serum-supplemented physiological buffer. This highlights the stabilizing effect of cisplatin cross-linking. In addition, the nanoparticles demonstrated excellent long-term stability, showing minimal changes in particle size and platinum content after two months of storage at 4 °C in aqueous solution. These findings suggest that cisplatin-mediated cross-linking provides a robust strategy for enhancing the structural stability of DNA-based nanomaterials without the need for complex DNA origami architectures.

Finally, the platinated DNA nanoparticles exhibited some level of radiosensitizing activity in FaDu cells. Compared to untreated controls and free DNA scaffold, the nanoparticles reduced the radiation dose required to achieve 10% survival, corresponding to a dose enhancement ratio of 1.17 ± 0.07. While the observed radiosensitization is moderate, this functionality provides a proof-of-concept for the design of stable DNA-based radiosensitizers. Further optimization may be achieved by systematically investigating parameters such as nanoparticle concentration, incubation time, cellular uptake, and cisplatin release strategies.

## Funding

The work was supported by the Ministry of Education, Youth and Sports of the Czech Republic, the European Union via the OP JAK project CZ.02.01.01/00/[0]22_008/0004558 “AMULET”, and the Czech Science Foundation project no. 24-11503S.

## Conflict of Interest

A patent application (PV 2025-491) partially based on this research has been filed at the Czech Industrial Property Office by the corresponding authors.

## Supporting Information

Supporting Information contains AGE/AFM micrographs of the thermal stability assay for the p7249 scaffold, an AGE micrograph of degradation assay in PBS with 10% FBS, the Pt content calibration of ICP-MS and UV–vis measurements, images of colonies evaluated for the clonogenic assay, clonogenic assays of cisplatin controls and cell plating efficiencies, and AFM micrographs of salt-less deposition of platinated DNA nanoparticles on mica.

File 1 Additional experimental data.

## Data Availability

Data generated and analyzed during this study is openly available in Zenodo at https://doi.org/10.5281/zenodo.21259520, reference number 21259520.
