# Supplementary material for: Facile synthesis of platinated DNA nanoparticles with radiosensitizing potential
Source: Beilstein J Nanotechnol. 2026 Aug 13;17:1117–27. doi: 10.3762/bjnano.17.76 (PMC13478894; doi:10.3762/bjnano.17.76)
Supplement: Supplementary file 1 [file Beilstein_J_Nanotechnol-17-1117-s001.pdf]

BEILSTEIN JOURNAL OF NANOTECHNOLOGY

Supporting Information

for

Facile synthesis of platinated DNA nanoparticles with radiosensitizing potential

Leo Sala, Tomas Perecko, Antonín Kaňa and Jaroslav Kočišek

Beilstein J. Nanotechnol. **2026,** *17,* 1117–1127. doi:10.3762/bjnano.17.76

Additional experimental data

License and Terms: This is a supporting information file under the terms of the Creative Commons Attribution License (https://creativecommons.org/licenses/by/4.0). Please note that the reuse, redistribution and reproduction in particular requires that the author(s) and source are credited and that individual graphics may be subject to special legal provisions.
The license is subject to the *Beilstein Journal of Nanotechnology* terms and conditions: (https://www.beilstein-journals.org/bjnano/terms)

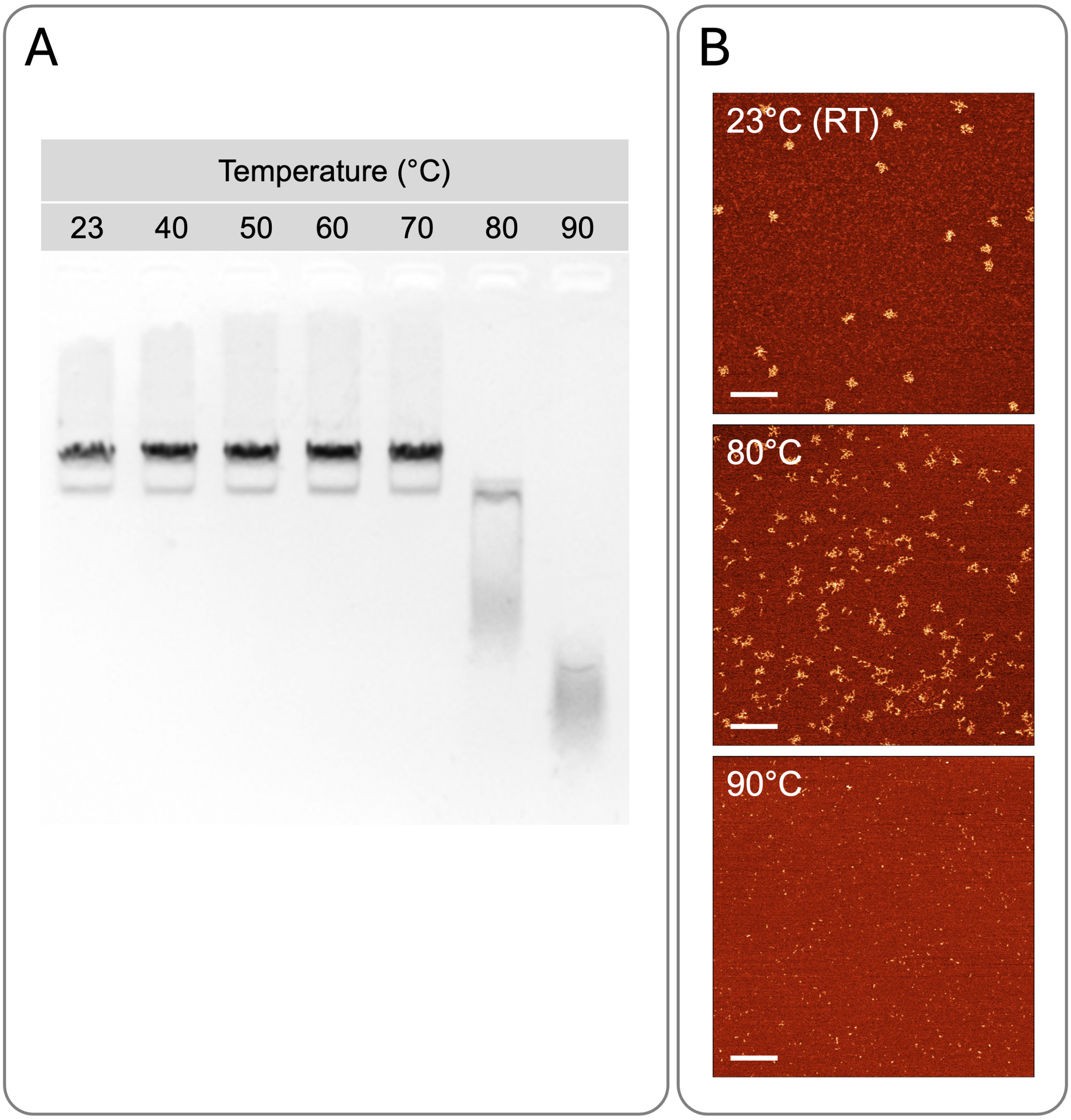

Figure S1: Thermal stability assay for the p7249 scaffold. AGE micrograph of p7249 scaffold solutions heated to different temperatures from room temperature to 90 °C (A) and AFM images of the p7249 scaffold samples at room temperature and those heated to 80 and 90 °C showing thermal degradation (B, scale bar = 400 nm, deposited on mica without additional buffer or salts).

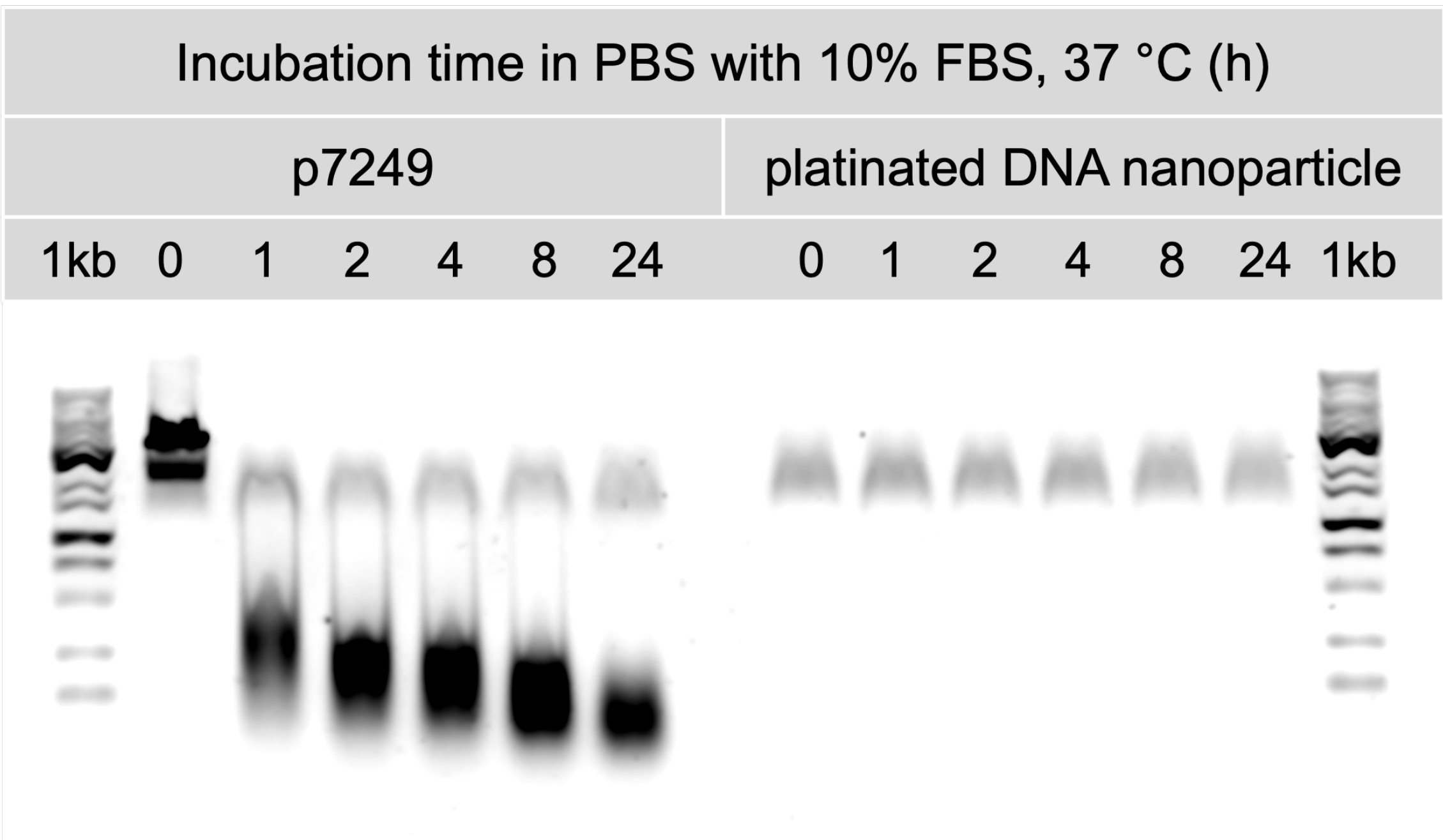

Figure S2: Stability of the p7249 scaffold and platinated DNA nanoparticles in physiological-like buffer. AGE micrograph of p7249 scaffold and platinated DNA nanoparticle solutions in PBS (pH 7.4) with 10 % FBS at 37 °C at different incubation times (0–24 h).

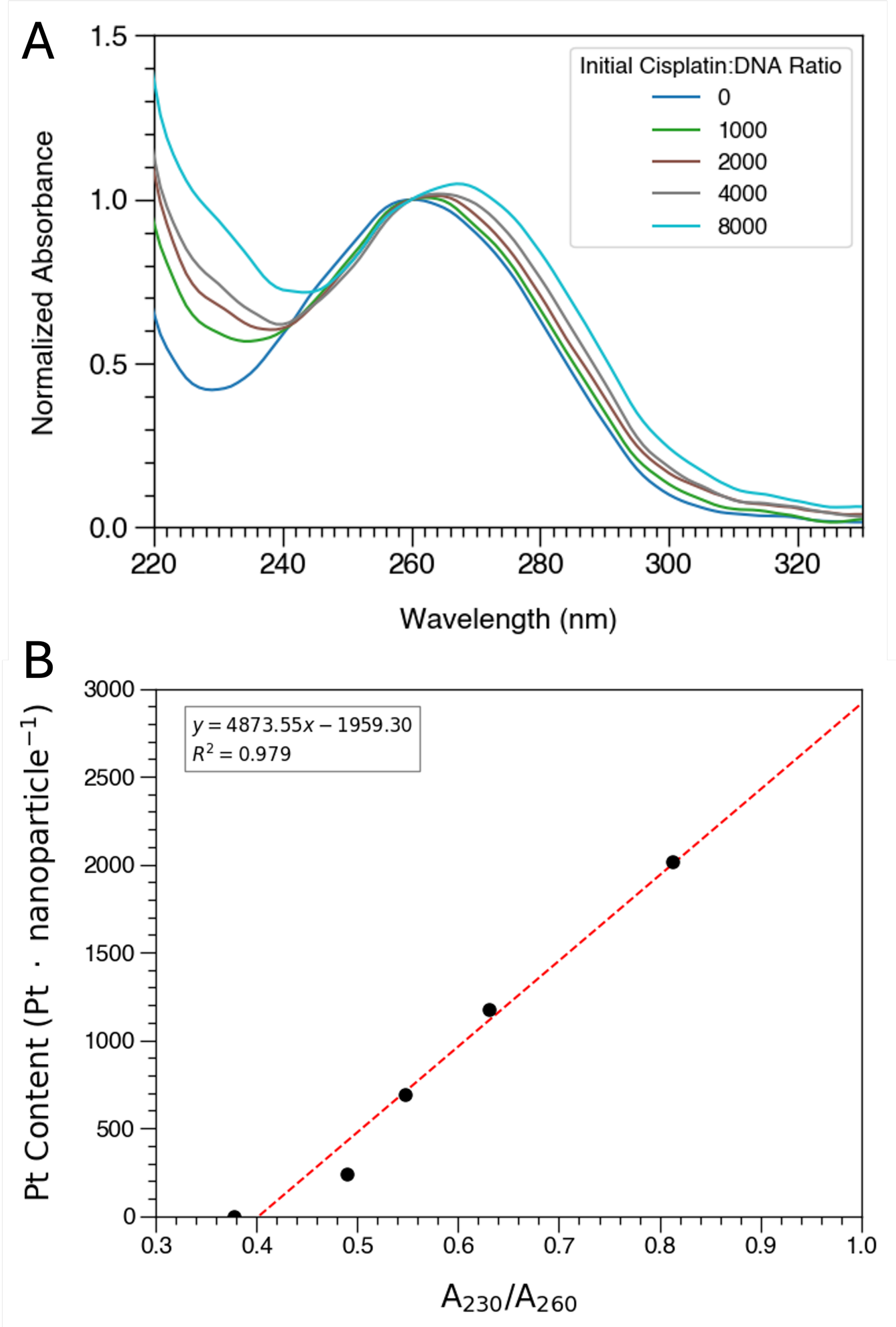

Figure S3: Correlation of Pt content and UV–vis spectra of platinated DNA nanoparticles. UV–vis spectra of filtered platinated DNA nanoparticles synthesized with different initial cisplatin/DNA scaffold ratios (A) and a plot of the measured Pt content from ICP-MS vs the ratio of absorbance values at 230 and 260 nm (A_{230}/A_{260}) fitted with a simple regression model to construct a calibration curve for Pt content estimations (B).

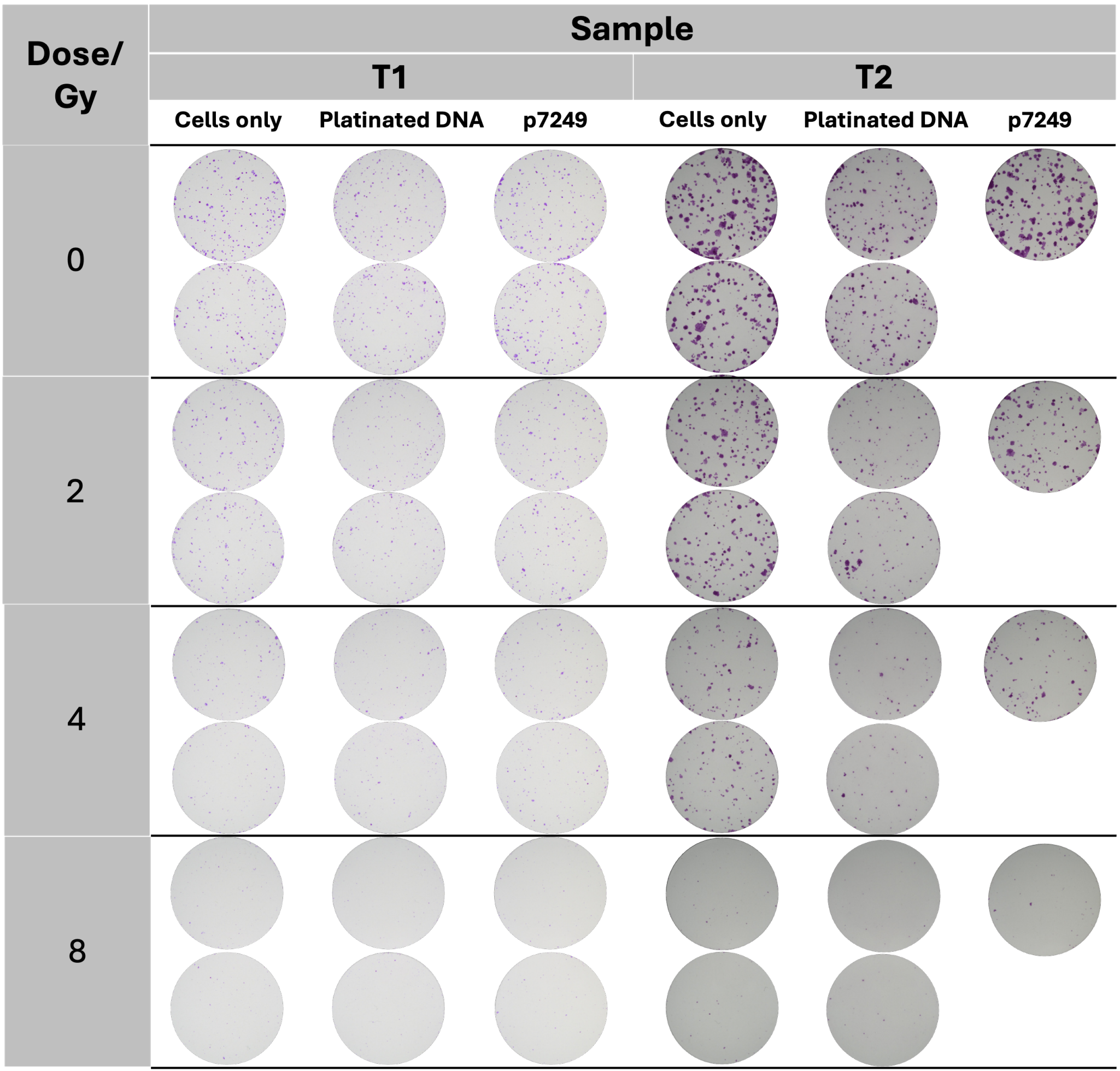

Camera and image analysis settings

Parameters	Values	
	T1	T2
Aperture	5.5 - 11	5.5 - 11
Light Source	Off	Off
White Balance	Off	Off
ImageJ Autothreshold	185-189	127-136
Colony Size Threshold	20-inf	20-inf
Circularity	0.1-1.0	0.1-1.0

Figure S4: Clonogenic assay. Images of stained cell colonies incubated with pure cell media (Cells only control), platinated DNA nanoparticles, and p7249 scaffold after the irradiation treatments. Camera and image analysis parameters for colony counting are tabulated in the lower panel.

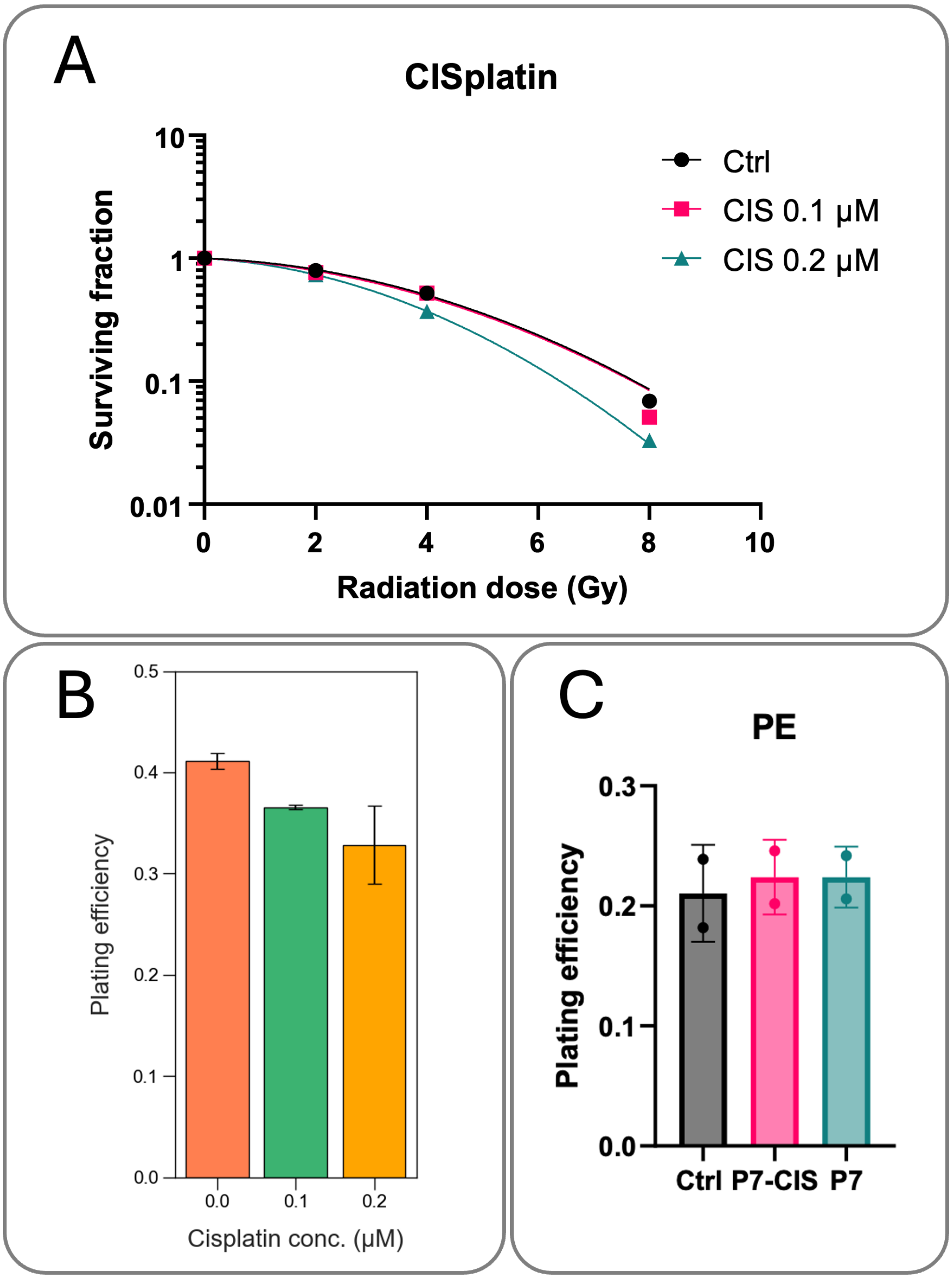

Figure S5: Clonogenic assay and plating efficiencies for cisplatin treatments. (A) Clonogenic assay results for two different concentrations of pure cisplatin (0.1 and 0.2 µM) compared to the control (cells only). (B) Plating efficiencies for pure cisplatin (0.1 and 0.2 µM, error bars indicate the standard deviation of technical duplicates). (C) Mean plating efficiencies of cells only (CTRL), platinated DNA nanoparticles (P7-CIS) and the pure ssDNA scaffold (P7), with error bars representing the standard deviation of two independent experiments.

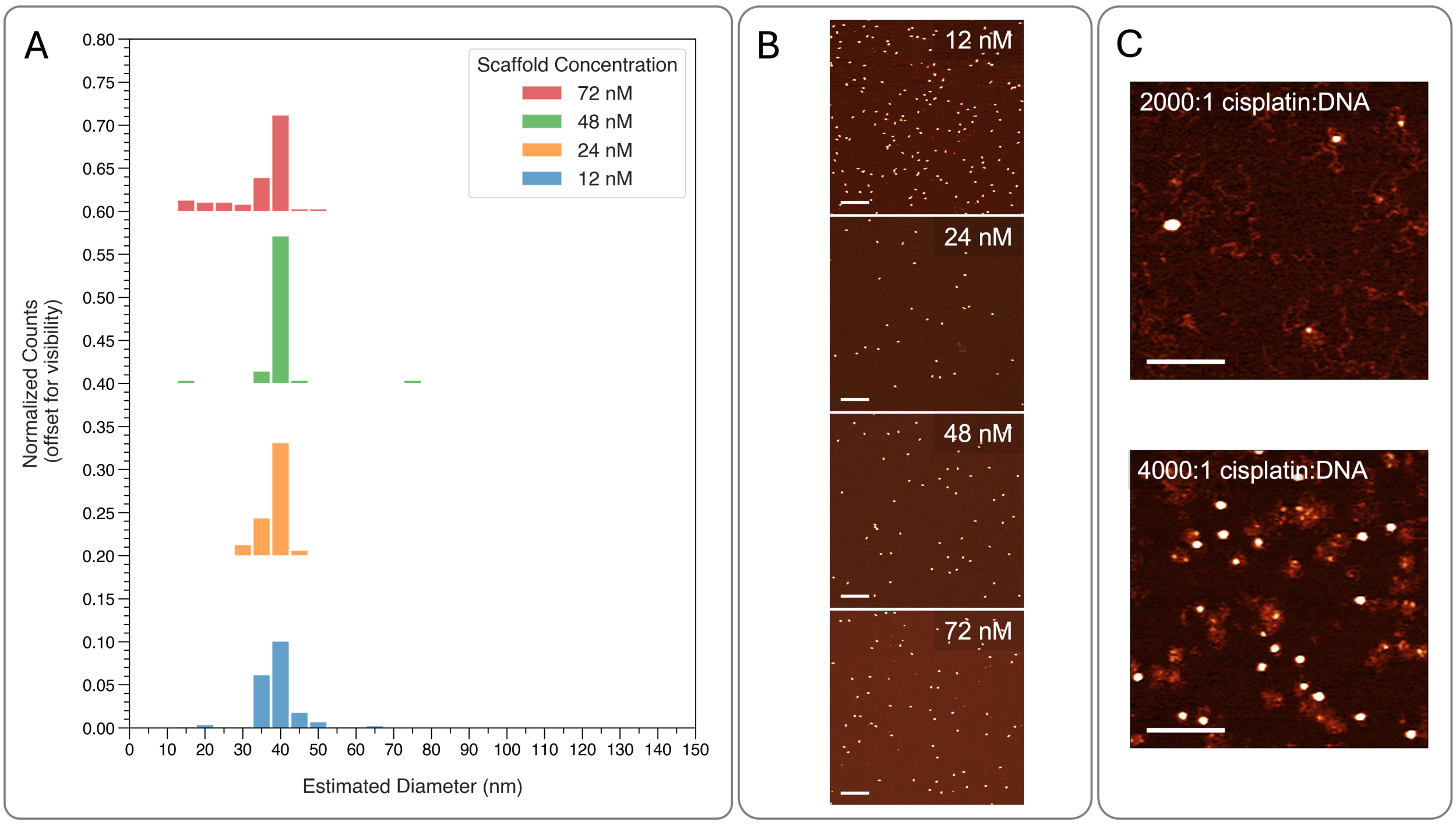

Figure S6: AFM characterization of platinated DNA nanoparticles on mica. Particle size distribution (A) and the corresponding AFM images (B) of the salt-free deposition of platinated DNA nanoparticles on mica synthesized with 8000:1 initial cisplatin/DNA ratio and with different scaffold concentrations (scale bar = 400 nm). AFM images of particles synthesized with lower initial cisplatin/DNA ratio (C) are shown to highlight partial, less compact cross-linking of the DNA scaffold (scale bar = 250 nm).
